# Shoutai Wan regulates maternal-fetal immune tolerance in lactate dehydrogenase A knockdown model mice to prevent miscarriage

**DOI:** 10.3389/fimmu.2026.1885151

**Published:** 2026-09-09

**Authors:** Ziwei Zhou, Yuhan Du, Ruigan Shi, Siling Tang, Mengmeng Zhou, Huilan Du

**Affiliations:** 1 Graduate School, Hebei University of Chinese Medicine, Shijiazhuang, China; 2 Collaborative Innovation Center of Integrated Chinese and Western Medicine on Reproductive Disease, Hebei University of Chinese Medicine, Shijiazhuang, China; 3 Hebei Key Laboratory of Integrative Medicine on Liver‑kidney Patterns, Hebei University of Chinese Medicine, Shijiazhuang, China; 4 College of Integrated Traditional Chinese and Western Medicine, Hebei University of Chinese Medicine, Shijiazhuang, China

**Keywords:** immune tolerance, maternal-fetal interface, lactate dehydrogenase A, Shoutai Wan, recurrent spontaneous abortion

## Abstract

**Problem:**

Maternal-fetal immune tolerance is vital for a healthy pregnancy. However, the metabolic factors governing this balance are not yet clear. This study investigates the role of LDHA in this process and explores how Shoutai Wan (STW) regulates uterine immune cells through LDHA.

**Methods:**

A uterine-specific LDHA-knockdown model was established by injecting AAV9-*Ldha*-RNAi into the uterus of CBA/J mice. Following STW pretreatment, female mice were mated with male BALB/c mice. Gravid uteri were collected on gestational days 6.5, 10.5, and 14.5. Embryo counts were recorded, and tissue morphology was observed using HE staining. Uterine immune cell populations including CD4^+^ T cells, CD8^+^ T cells, M1/M2-like macrophages, Th1/Th2 cells, Tregs, and NK cells were analyzed by immunohistochemistry and flow cytometry. IL-4, Foxp3, IFN-γ, and TGF-β1 expression were quantified by qRT-PCR and Western blot.

**Results:**

Uterine LDHA knockdown decreased embryo number and disrupted maternal-fetal interface morphology. STW treatment reversed these effects. IHC showed that STW increased CD4^+^ T cells, F4/80^+^ macrophages, and NK cells at the maternal-fetal interface of sh-*Ldha* mice and reduced CD8^+^ T cell infiltration. Flow cytometry further revealed that STW increased the absolute numbers of uterine Tregs and NK cells in early and mid‑pregnancy, whereas reducing Th1 cell counts at all three time points. Both Western blot and qRT‑PCR analyses confirmed that STW upregulated the protein and mRNA expression of LDHA, IL‑4, TGF‑β1, and Foxp3, while suppressing that of IFN‑γ at the maternal‑fetal interface.

**Conclusion:**

Uterine LDHA inhibition disrupts maternal-fetal immune tolerance in pregnant CBA/J mice. STW treatment restores this balance in sh-*Ldha* mice, supporting a successful pregnancy.

## 1 Introduction

Recurrent spontaneous abortion (RSA), or recurrent pregnancy loss (RPL), is a major reproductive complication affecting approximately 1–5% of couples attempting conception and is generally defined as two or more consecutive pregnancy losses before 24 weeks of gestation (1–3). Although chromosomal abnormalities, endocrine disorders, and uterine malformations are recognized etiologies, more than half of cases remain unexplained and are classified as unexplained recurrent pregnancy loss (URPL) (4). Increasing evidence indicates that aberrant maternal-fetal immune tolerance is closely associated with URPL, highlighting the importance of immune homeostasis at the maternal-fetal interface (5, 6). Successful pregnancy requires the maternal immune system to tolerate fetal alloantigens while maintaining appropriate immune surveillance. This process is coordinated by multiple decidual immune cell populations, including decidual natural killer (dNK) cells, decidual macrophages (dMφ), and T lymphocytes (7–13). During pregnancy, appropriate M2-like macrophage polarization and regulatory T (Treg) cell activity contribute to a tolerant maternal-fetal environment, whereas excessive pro-inflammatory M1-like and Th1 responses are associated with pregnancy failure (14–22).

Metabolic reprogramming at the maternal-fetal interface is increasingly recognized as an important regulator of pregnancy and immune homeostasis. Trophoblasts rely substantially on aerobic glycolysis to meet their high energy and biosynthetic demands (23–25). Impaired glycolytic activity has been associated with defective trophoblast proliferation and invasion, abnormal decidualization, and adverse pregnancy outcomes in RSA models (26–29). Lactate dehydrogenase A (LDHA), which catalyzes the conversion of pyruvate to lactate, is a key enzyme in this metabolic pathway. Lactate produced through glycolysis can modify the local microenvironment and influence immune-cell function, including macrophage polarization (23, 30). Consistent with this metabolic-immune link, reduced uterine aerobic glycolysis has been reported in RSA, suggesting that disruption of LDHA-associated metabolism may contribute to impaired maternal-fetal immune tolerance (26). However, the relationship between uterine LDHA and immune-cell homeostasis during pregnancy remains insufficiently characterized.

Shoutai Wan (STW), a classical Chinese herbal formula, has long been used for the prevention of recurrent miscarriage and has been reported to improve endometrial receptivity, promote decidualization, and regulate the uterine immune microenvironment (31–36). Our previous studies demonstrated that STW restores impaired uterine aerobic glycolysis and improves fetal survival in RSA mice (27, 31). Moreover, previous work has suggested that STW can modulate immune-related factors and T-cell immune tolerance at the maternal-fetal interface (34, 35). However, whether LDHA serves as a metabolic regulator linking STW treatment to maternal-fetal immune homeostasis remains unclear. In particular, it is unknown whether suppression of uterine LDHA is sufficient to induce immune-cell and cytokine abnormalities resembling the pro-inflammatory state observed in RSA, and whether STW can reverse these changes.

In the present study, we established a uterine-specific LDHA knockdown mouse model and evaluated pregnancy outcomes, maternal-fetal interface morphology, immune-cell populations, and immune-related cytokines at GD 6.5, 10.5, and 14.5. We hypothesized that uterine LDHA deficiency disrupts maternal-fetal immune homeostasis, characterized by enhanced pro-inflammatory M1-like/Th1 responses and reduced M2-like/Th2/Treg/NK populations, thereby compromising pregnancy outcomes. We further hypothesized that STW restores LDHA expression and alleviates this immune imbalance, thereby improving maternal-fetal immune tolerance and pregnancy maintenance. By integrating metabolic and immunological analyses, this study aimed to clarify the role of LDHA in maternal-fetal immune regulation and provide mechanistic insight into the therapeutic effects of STW in RSA.

## 2 Materials and methods

### 2.1 Animals and ethics statement

Specific pathogen-free female CBA/J mice (7–8 weeks, 18–20 g, n = 180) and male BALB/c mice (9–10 weeks, 25–30 g, n = 90) were obtained from Beijing HFK Bioscience Co., Ltd. (SCXK [Beijing] 2019-0008). All procedures were approved by the Ethics Committee of Hebei University of Chinese Medicine (No. DWLL2021089). Mice were housed under controlled conditions (22-25 °C, 40%-60% humidity, 12-h light/dark cycle) with freely access to food and water. Animals were acclimated for 7 days prior to experimental initiation.

### 2.2 AAV9-*Ldha*-RNAi virus preparation and modeling

CBA/J mice (n = 180) were randomly assigned to four groups (n = 45/group): Saline, sh-Ctrl, sh-*Ldha*, and sh-*Ldha*+STW. A uterine-specific *Ldha*-knockdown mouse model was generated using AAV9-sh*Ldha* (Vector GV478, U6-MCS-CAG-EGFP; 4.18 × 10^13^ v.g/mL; GeneChem, China). Mice were anesthetized with tribromoethanol (0.2 mL/10 g; Dowobio, China). Following a longitudinal dorsal incision, the uterine horns were exteriorized, and AAV9-sh*Ldha* or negative control virus (AAV9-CON305) was administered via intra-uterine horn injection (1 × 10^11^ v.g in 10 μL saline per horn) using a microsyringe (37). Post-surgical recovery was monitored before subsequent interventions.

### 2.3 Preparation of Shoutai Wan decoction and administration protocol

The constituents of STW (**Supplementary Table 2**): *Cuscutae Semen*, *Taxilli Herba*, *Dipsaci Radix*, and *Asini Corii Colla* were authenticated according to the Chinese Pharmacopoeia (2020) or the Medicinal Plant Names Service (MPNS) and prepared as a decoction with a final concentration of 1.5 g/mL. Following the Traditional Chinese Medicine principle of “pre-treatment of impairment” (Yu Pei Qi Sun), intervention should be initiated during the preconception and early pregnancy periods before embryonic damage occurs. Human secondary follicle development into early antral follicles takes about 85–90 days (38); thus, a three-month pre-treatment period can effectively improve oocyte quality. The murine estrous cycle lasts 4–5 days; therefore, a 14-day administration period covers approximately three complete cycles, corresponding to the clinical treatment duration in humans.

Female CBA/J mice in the sh-*Ldha*+STW group began receiving medication on the 8th day post-surgery. Based on our previous study (27), the optimal dosage of STW was selected as twice the human clinical equivalent dose (0.15 g/10 g/day). Control groups received distilled water via gavage (0.1 mL/10 g/day). Gavage was performed daily every morning for 14 consecutive days. Following 14 days of administration, female CBA/J mice were co-caged with male BALB/C mice (2:1). Detection of a vaginal plug the next morning was designated as gestational day (GD) 0.5, and daily dosing continued throughout the pregnancy. On GD 6.5, 10.5, and 14.5, 15 mice per group were randomly selected and anesthetized with tribromoethanol. Following sacrifice by cervical dislocation, gravid uteri were harvested. Fresh tissues were processed as follows: one portion was immersed in pre-cooled saline for flow cytometry, while another was fixed in 4% paraformaldehyde for HE or IHC staining. For the remaining samples, embryos were removed, and the decidua or placenta (including the attached uterine wall) were collected and stored at -80 °C for Western blot and qRT-PCR analysis. The complete flowchart for the animal experiments is presented in **Figure 2A**.

### 2.4 Embryo counting

Gravid uteri were rinsed with PBS to record total implantation and resorption sites. Normal embryos were defined by a translucent appearance and uniform size, while resorbed sites showed hemorrhage, reduced volume, and lack of identifiable fetal structures.

### 2.5 Hematoxylin-Eosin staining and immunohistochemical saining

Fresh gravid uteri were fixed in 4% paraformaldehyde, dehydrated, and embedded in paraffin. Transverse sections (4 μm thick) were prepared and stained with hematoxylin and eosin to observe morphological changes at the maternal-fetal interface under an optical microscope. For immunohistochemistry, sections were deparaffinized, rehydrated, and subjected to heat-induced antigen retrieval (pH 9.0), then incubated overnight at 4 °C with primary antibodies against CD4, CD8α, F4/80, CD49a, and CD49b. Decidual macrophages were identified by F4/80 expression (39), NK cell subsets were distinguished using CD49a for tissue-resident cells and CD49b for peripherally recruited conventional NK cells (40–42). Signal detection was performed using an HRP-conjugated secondary polymer system and DAB chromogen, followed by hematoxylin counterstaining. Immunoreactivity and cellular morphology at the maternal-fetal interface were observed by light microscopy. Antibody and reagent details are in **Supplementary Tables 3**, **4**.

### 2.6 Flow cytometry analysis

Decidual and placental single-cell suspensions were prepared via mechanical dissociation and enzymatic digestion (0.125% trypsin). The following gating strategies were applied for each immune subset. For all populations, cells were first selected based on morphology (FSC-H/SSC-H) to enrich intact cells, followed by doublet discrimination (FSC-H/FSC-A). Dead cells were excluded based on low FSC and low SSC signals, without the use of a commercial viability dye.

For macrophage polarization, cells were surface-stained for CD86, then fixed, permeabilized, and intracellularly labeled for iNOS and CD206. The gating strategy followed: main population → single cells → M1-like (CD86^+^iNOS^+^) and M2-like (CD206^+^CD71^+^) subsets. It should be noted that due to the absence of a prior macrophage-lineage marker (e.g., F4/80/CD11b) in this panel, the populations identified are referred to as M1-like and M2-like cells rather than definitive M1/M2 macrophages, and this limitation is addressed in the Discussion. For T cell subsets, after main population and single-cell selection, CD3^+^ T lymphocytes were gated. To evaluate T cell subsets, cells were stimulated for 4 h with a cell stimulation cocktail. Within CD3^+^CD4^+^ cells, Th1 and Th2 cells were identified as IFN-γ^+^ and IL-4^+^, respectively. Treg cells were defined as CD3^+^CD4^+^CD25^+^Foxp3^+^ using a transcription factor buffer set. NK cells were identified as NKp46^+^ within the CD3^−^ population.

All flow cytometry data were acquired on an Attune NXT flow cytometer (Invitrogen, USA) and analyzed using appropriate compensation controls, isotype controls, and single-stained controls. Antibody and reagent details are in **Supplementary Tables 3**, **4**. The detailed gating hierarchies for each immune subset are summarized in **Supplementary Table 6**.

Dead cells were excluded based on low FSC/SSC signals without the use of a commercial viability dye.

### 2.7 Western blot and quantitative real-time reverse transcription polymerase chain reaction

Total protein was extracted from maternal-fetal interface tissues and quantified using a BCA assay. Equal amounts of protein were separated by 12.5% SDS-PAGE and transferred onto PVDF membranes. After blocking with 5% non-fat milk, membranes were incubated at 4 °C overnight with primary antibodies against LDHA, TGF-β1, Foxp3, IL-4, and IFN-γ. β-actin or β-tubulin served as internal controls. Following incubation with HRP-conjugated secondary antibodies, protein bands were visualized via enhanced chemiluminescence and quantified using ImageJ software. Total RNA was isolated from uterine tissues and reverse-transcribed into cDNA. qRT-PCR was performed on an AB 7500 Fast Real-Time PCR system. Relative mRNA expression levels of target genes were normalized to internal controls and calculated using the 2^- ΔΔCT^ method. The primer sequences used for qRT-PCR are summarized in **Supplementary Table 5**. Antibody and reagent details are in **Supplementary Tables 3, 4**.

### 2.8 Blinding and randomization

Prior to the experiment, 180 female CBA/J mice were randomly allocated into four groups (Saline, sh-Ctrl, sh-*Ldha*, and sh-*Ldha*+STW) using a random number table generated by Excel. Within each group, mice were sequentially ordered by random numbers and assigned to one of three gestational time points (GD 6.5, 10.5, and 14.5), with 15 mice per group per time point. All animals were identified by ear tags bearing numerical codes only, with no group information. Viral injection solutions (saline, control AAV9, and AAV9-sh*Ldha*) were coded as A, B, and C in identical EP tubes by an independent investigator who was not involved in animal procedures or data collection. Surgeries, gavage administration, mating observation, and tissue collection were performed by two investigators, who were blinded to group allocation throughout the experimental procedures. Tissue samples were labeled solely with animal codes and gestational time points. All downstream assays (histology, immunohistochemistry, flow cytometry, Western blot, and qRT-PCR) were performed by investigators blinded to group identity. The code was revealed only after all data acquisition and analyses were completed.

### 2.9 Statistical analysis

Data are presented as mean±SEM. Normality was assessed using the Shapiro-Wilk test. For normally distributed data, variance homogeneity was evaluated by the Brown-Forsythe test. Groups were compared using one-way ANOVA followed by Tukey’s multiple comparisons test; in cases of unequal variances, Welch’ s ANOVA was applied. Non-normally distributed data were analyzed using the Kruskal-Wallis test. All analyses were performed using GraphPad Prism 10.1.2 (GraphPad Software, USA). A P-value < 0.05 was considered statistically significant. Significance levels are denoted as: *P < 0.05, **P < 0.01, ***P < 0.001, and ****P < 0.0001.

Sample sizes were determined based on our previous studies in RSA mouse models (27, 31), in which a 30–40% reduction in embryo number was observed in mice with impaired uterine glycolysis. With α = 0.05 and statistical power of 0.80, a minimum of 12 mice per group per time point was required. To account for potential surgical failure, mating failure, and embryonic resorption, 15 mice per group per time point were included.

## 3 Results

### 3.1 Validation of Uterine-specific LDHA knockdown by AAV9-shRNA

**Figure 1:**
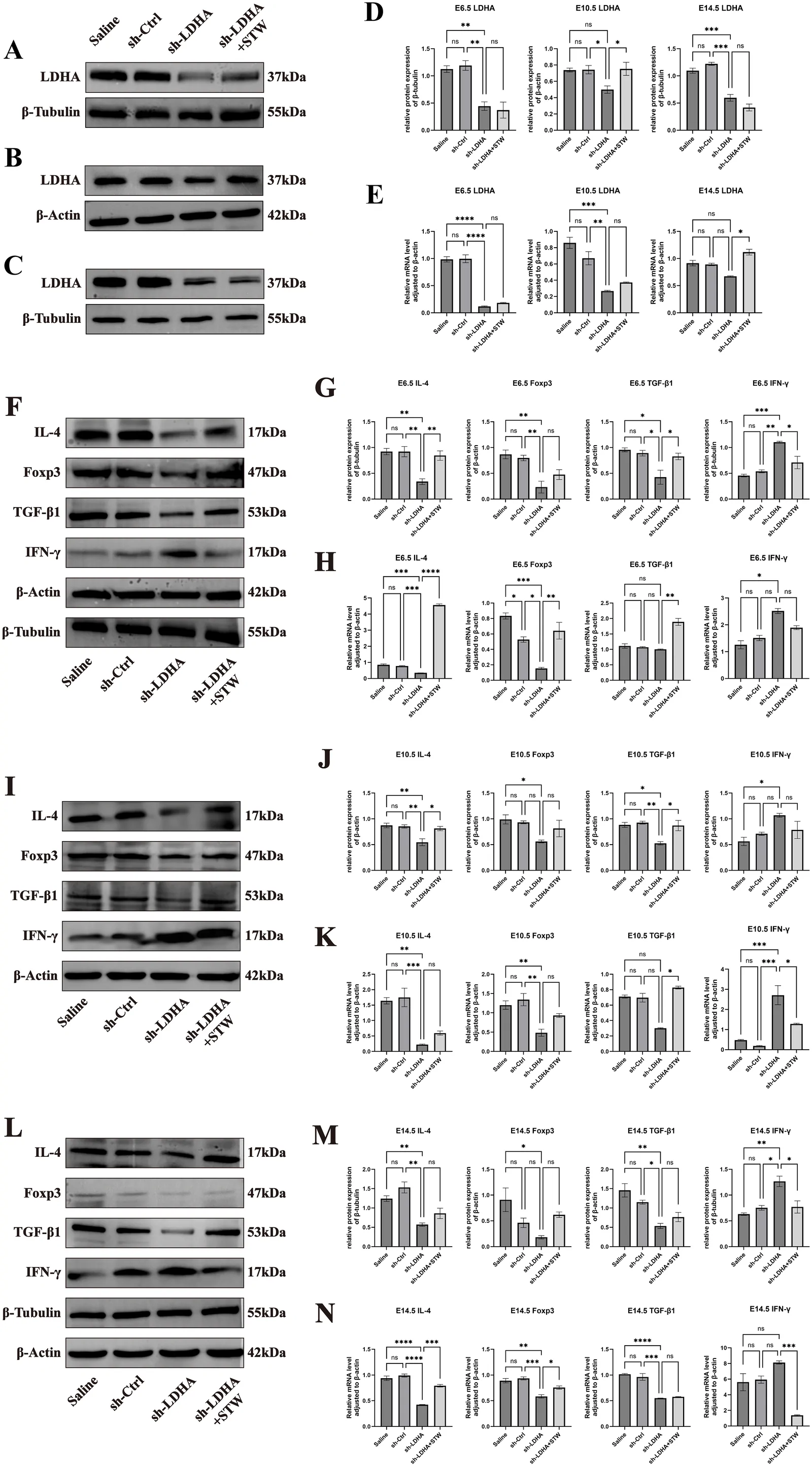
STW modulates LDHA and cytokine expression at the maternal-fetal interface across gestation. **(A–C)** Effects of STW on protein levels of LDHA at the maternal-fetal interface on GD 6.5, 10.5, and 14.5. **(D)** Statistical analysis of LDHA protein expression levels at the maternal-fetal interface on GD 6.5, 10.5, and 14.5 across all groups. **(E)** Effects of STW on mRNA levels of LDHA at the maternal-fetal interface. **(F, I, L)** Effects of STW on protein levels of IL-4, Foxp3, TGF-β1, and IFN-γ at the maternal-fetal interface on GD 6.5, 10.5, and 14.5. **(G, J, M)** Statistical analysis of protein expression levels for IL-4, Foxp3, TGF-β1, and IFN-γ at the maternal-fetal interface on GD 6.5, 10.5, and 14.5 across all groups. **(H, K, N)** Effects of STW on mRNA levels of IL-4, Foxp3, TGF-β1, and IFN-γ at the maternal-fetal interface on GD 6.5, 10.5, and 14.5. Quantitative data are presented as mean ± SEM. n = 3 biologically independent samples per group for Western blot and qRT-PCR. *P < 0.05; **P < 0.01; ***P < 0.001; ****P < 0.0001. Quantitative data are presented as mean ± SEM. The symbol "ns" denotes not significant.

To simulate inhibited aerobic glycolysis at the maternal-fetal interface in RSA mice, a uterine-specific *Ldha*-knockdown model was generated via AAV9-shRNA. LDHA mRNA and protein expression at the maternal-fetal interface was significantly reduced in sh-*Ldha* mice across all three gestational time points compared to controls. STW treatment partially restored LDHA expression, counteracting its inhibition at the maternal-fetal interface (**Figures 1A–E**). This established a reliable model for analyzing subsequent immune cell populations.

### 3.2 STW increases the number of embryos in pregnant sh-*Ldha* mice

**Figure 2:**
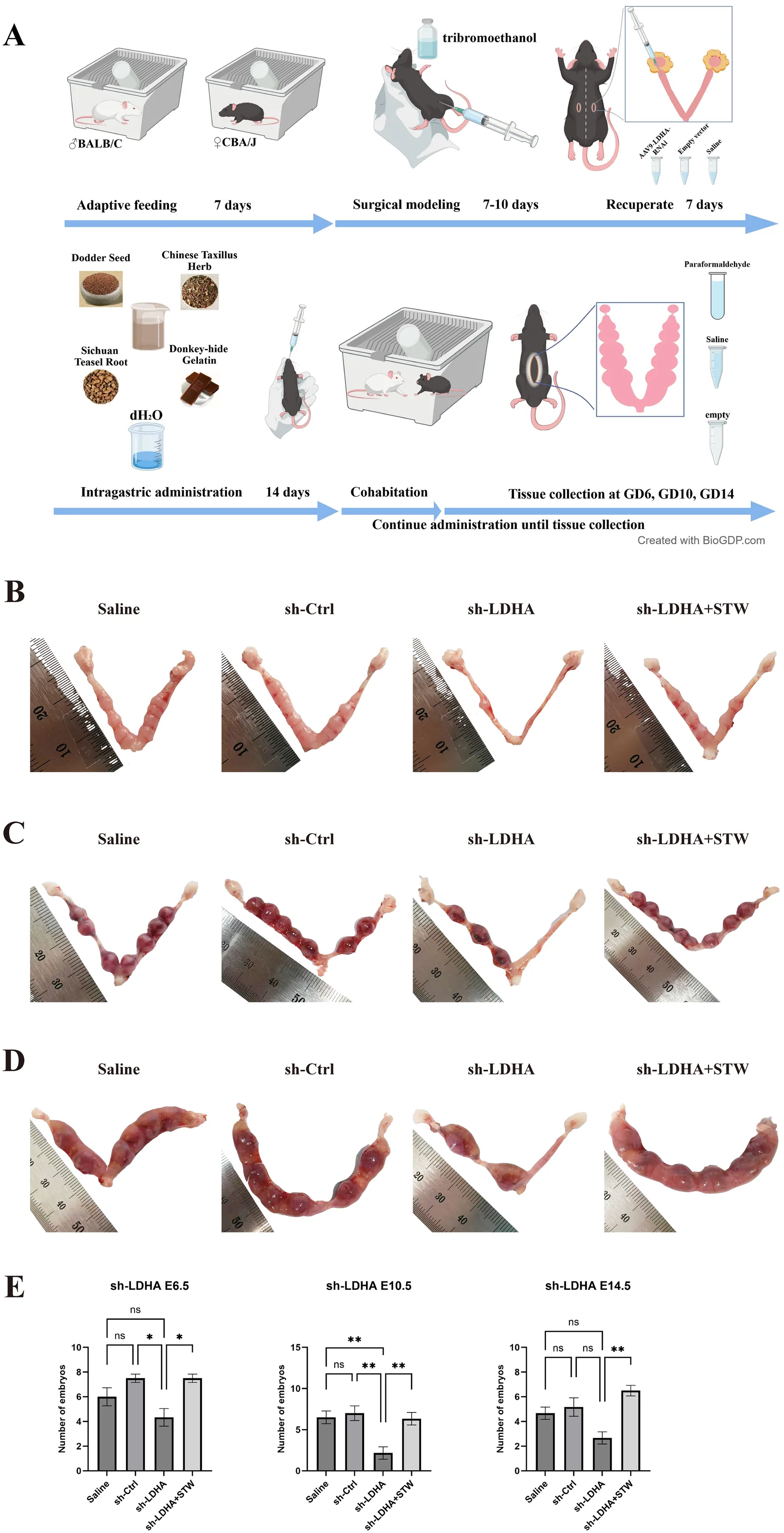
STW increases embryo numbers in sh-*Ldha* mice. **(A)** Experimental flowchart [created using BioGDP.com (43)]. **(B–D)** Representative images showing STW’ s effect on embryo numbers at GD 6.5, 10.5, and 14.5. **(E)** Quantitative analysis of embryo counts across groups. Data are presented as mean ± SEM. n = 6 mice per group per time point for embryo counts. *P < 0.05; **P < 0.01. The symbol "ns" denotes not significant.

To evaluate the effect of uterine LDHA inhibition on pregnancy outcomes, we recorded the number of embryos in each group. Compared to the saline and sh-Ctrl groups, sh-*Ldha* mice showed a significant reduction in the number of embryos at GD 6.5 and 10.5. These reductions were effectively reversed by STW treatment. At GD 14.5, although embryo counts in the sh-*Ldha* group were comparable to controls, STW treatment significantly increased embryo numbers relative to the sh-*Ldha* group. No significant differences were observed between the saline and sh-Ctrl groups throughout the study (**Figures 2B–E**).

### 3.3 STW restored maternal-fetal tissue architecture in sh-*Ldha* mice

**Figure 3:**
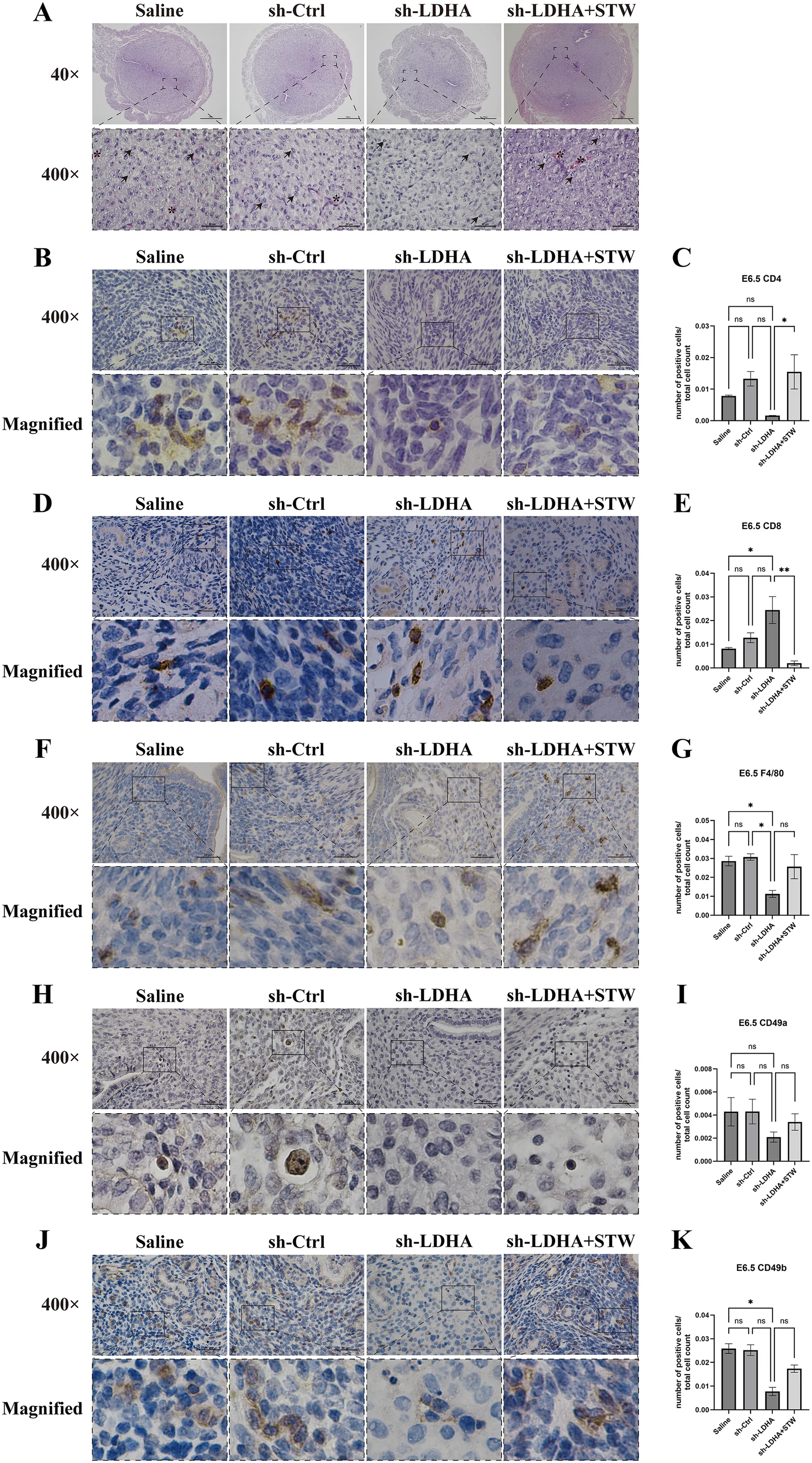
STW maintains placental architecture and modulates immune cell infiltration at GD 6.5. **(A)** Representative HE staining of the gravid uterus (top: 40 × magnification, scale bar = 500 μm) and decidual cells (bottom: 400 × magnification, scale bar = 50 μm) at GD 6.5. Arrows (→) indicate typical decidual cells; asterisks (*) denote decidual capillary lumens. **(B–K)** Immunological profiling of the decidua at GD 6.5. Representative IHC staining **(B, D, F, H, J)** and corresponding quantitative analysis **(C, E, G, I, K)** are shown for CD4^+^ T cells, CD8^+^ T cells, F4/80^+^ macrophages, and NK cell subsets (CD49a^+^ trNK and CD49b^+^ cNK). For IHC images (top: 400× magnification), scale bar = 50 μm. Bottom panels show magnified views of representative areas. Data are expressed as mean ± SEM. n = 3 randomly selected fields per group for IHC quantification. * *P* < 0.05, ** *P* < 0.01. The symbol "ns" denotes not significant.

To further observe histological changes at the maternal-fetal interface after uterine LDHA inhibition, HE staining was performed on transverse uterine sections. On GD 6.5, uterine stromal cells in the Saline and sh-Ctrl groups successfully transformed into mature decidual cells. These cells were large, nutrient-rich, and featured prominent nucleoli with characteristic “vacuolated” cytoplasm. Histology showed dense cell clusters and a well-developed capillary network. However, sh-*Ldha* mice exhibited impaired decidualization, marked by fewer and smaller decidual cells, faint nucleoli, and minimal vacuolation. This group also showed sparse capillary growth and poor blood supply, signaling delayed decidual progress. STW treatment reversed these impairments, promoting decidual cell maturation and restoring vascular density (**Figure 3A**).

**Figure 4:**
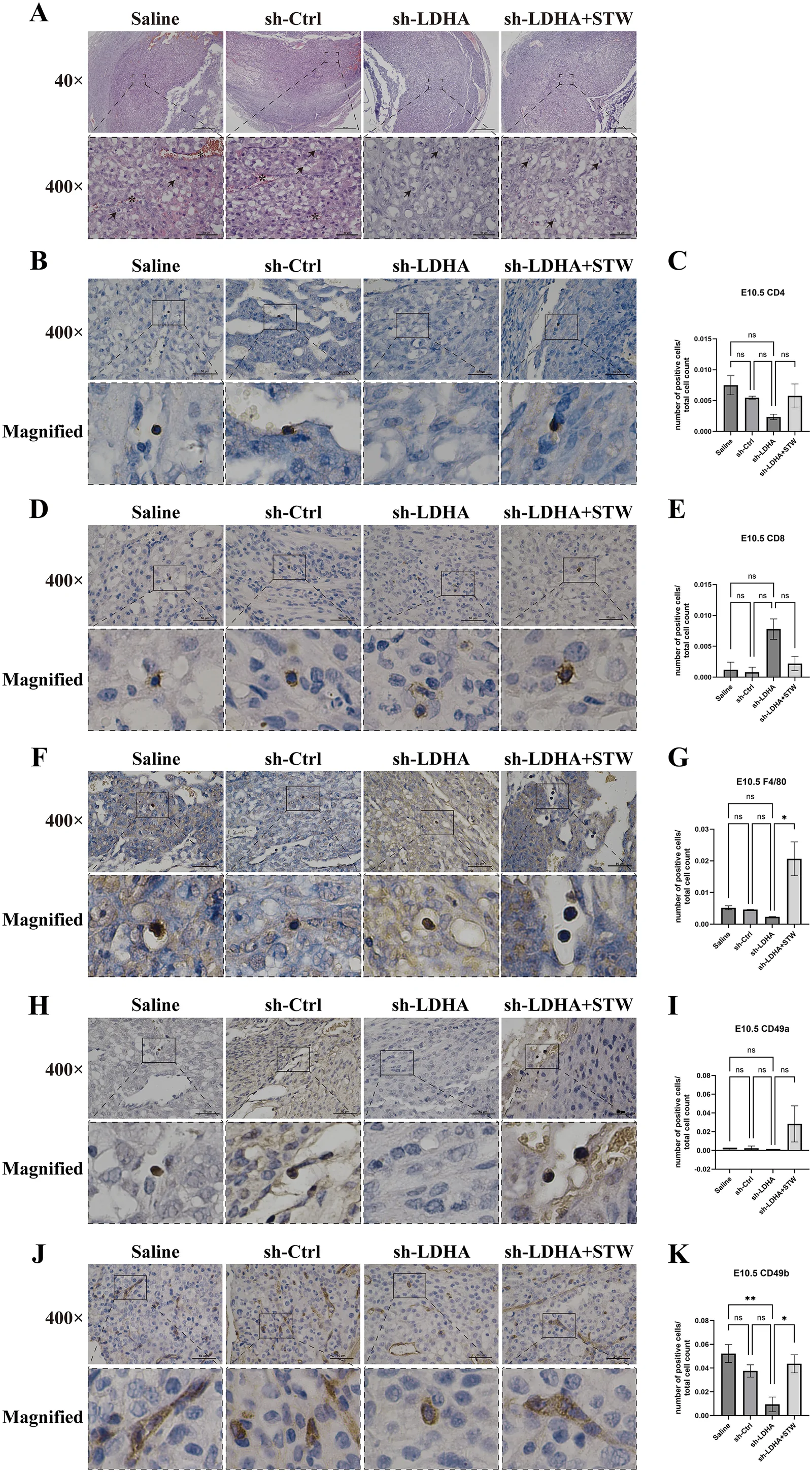
STW maintains placental architecture and modulates immune cell infiltration at GD 10.5. **(A)** Representative images of placental morphology at GD 10.5. Top panels provide an overview (40× magnification scale bar = 500 μm), while bottom panels show high-magnification views (400× magnification scale bar = 50 μm). Arrows (→) indicate representative decidual cells; asterisks (*) denote fetal capillary lumens. **(B–K)** Immunological profiling of the decidua at GD 10.5. Representative IHC images **(B, D, F, H, J)** and corresponding quantitative analysis **(C, E, G, I, K)** are provided for CD4^+^ T cells, CD8^+^ T cells, F4/80^+^ macrophages, and NK cell subsets (CD49a^+^ trNK and CD49b^+^ cNK). For IHC images (top), 400× magnification scale bar = 50 μm. Bottom panels show magnified views of representative areas. Data are expressed as mean ± SEM. n = 3 randomly selected fields per group for IHC quantification. * *P* < 0.05, ** *P* < 0.01. The symbol "ns" denotes not significant.

On GD 10.5, the placental labyrinth was in an early developmental phase. The Saline and sh-Ctrl groups exhibited high placental cellularity with a dense cell arrangement. Numerous decidual cells featured characteristic “vacuolated” cytoplasm, and maternal blood sinuses were filled with erythrocytes, indicating effective placental blood supply. In contrast, sh-*Ldha* mice lacked these blood-filled sinuses and showed a marked decrease in placental cell density, suggesting a compromised blood supply. STW intervention reversed these impairments, increasing decidual cell density and restoring blood flow within the placental region (**Figure 4A**).

**Figure 5:**
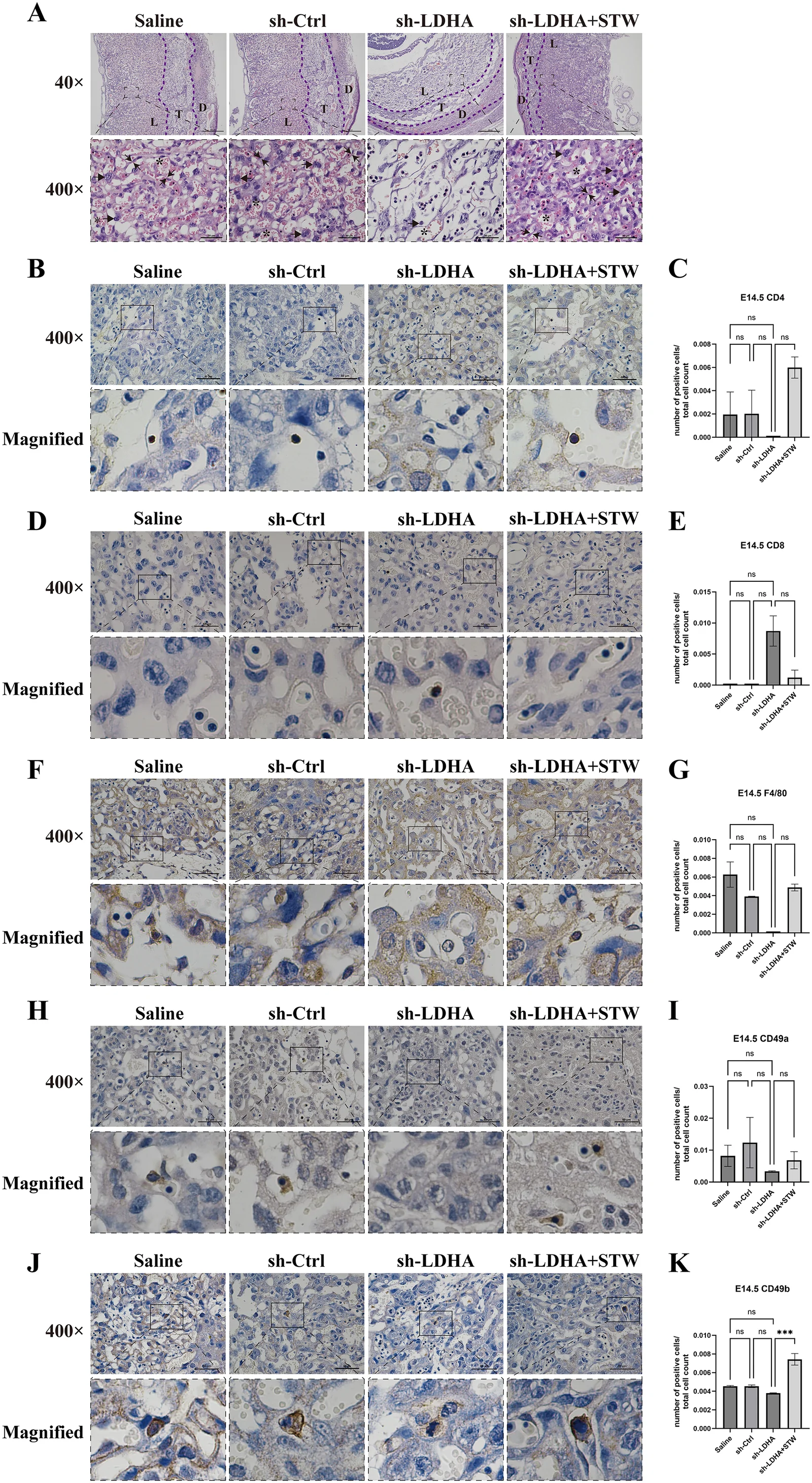
STW maintains placental architecture and modulates immune cell infiltration at GD 14.5. **(A)** Representative images of placental architecture at GD 14.5. Top panels provide an overview (40× magnification scale bar = 500 μm); bottom panels show high-magnification views of the labyrinth layer (400× magnification scale bar = 50 μm). L, labyrinth layer; T, trophoblast layer (junctional zone); D, decidual zone. Arrows (→←) indicate fetal-derived capillary walls; asterisks (*) denote maternal blood sinuses; arrowheads (▶) highlight representative trophoblast cells. **(B–K)** Immunological profiling of the decidua at GD 14.5. Representative IHC staining **(B, D, F, H, J)** and corresponding quantitative analysis **(C, E, G, I, K)** are shown for CD4^+^ T cells, CD8^+^ T cells, F4/80^+^ macrophages, and NK cell subsets (CD49a^+^ trNK and CD49b^+^ cNK). Top panels: 400× magnification scale bar = 50 μm. Bottom panels show magnified views of representative areas. Data are expressed as mean ± SEM. n = 3 randomly selected fields per group for IHC quantification. ***P < 0.001. The symbol "ns" denotes not significant.

By GD 14.5, control placentas showed clearly organized labyrinth, junctional, and decidual zones. The labyrinth contained dense villi, abundant trophoblasts with oval nuclei, and intact vessels filled with erythrocytes. In contrast, sh-*Ldha* mice had disorganized villi, fewer trophoblasts, and fragmented vascular walls with enlarged lumens and minimal blood cells. STW treatment corrected these defects, restoring compact villi and placental circulation to levels resembling healthy controls (**Figure 5A**).

### 3.4 STW modulates immune cell infiltration at the maternal-fetal interface

We further examined the distribution of major immune cell subsets at the maternal-fetal interface across all groups using immunohistochemistry. At GD 6.5, the proportion of decidual CD8^+^ T cells in the *sh-Ldha* group was significantly higher than in the Saline and sh-Ctrl groups. In contrast, the percentages of F4/80^+^ macrophages and CD49a^+^ NK cells were lower in *sh-Ldha* mice compared to controls. Following STW treatment, the proportion of CD4^+^ T cells increased, whereas CD8^+^ T cells decreased relative to the *sh-Ldha* group (**Figures 3B–K**).

On GD 10.5, CD49b^+^ NK cell counts in the *sh-Ldha* group were reduced compared to the Saline group. Notably, STW treatment significantly increased the proportions of both F4/80^+^ macrophages and CD49b^+^ NK cells. No significant differences in immune cell populations were observed between the Saline and sh-Ctrl groups (**Figures 4B–K**).

By GD 14.5, the proportions of CD4^+^ T cells, CD8^+^ T cells, F4/80^+^ macrophages, and CD49a^+^ NK cells showed no significant differences between the *sh-Ldha* and control groups. However, STW treatment significantly elevated the proportion of CD49b^+^ NK cells compared to the *sh-Ldha* group. No significant changes were found in CD4^+^, CD8^+^, or F4/80^+^ populations between the STW and *sh-Ldha* groups (**Figures 5B–K**).

### 3.5 STW modulates the immune cell counts in the gravid uterus of sh-*Ldha* mice

**Figure 6:**
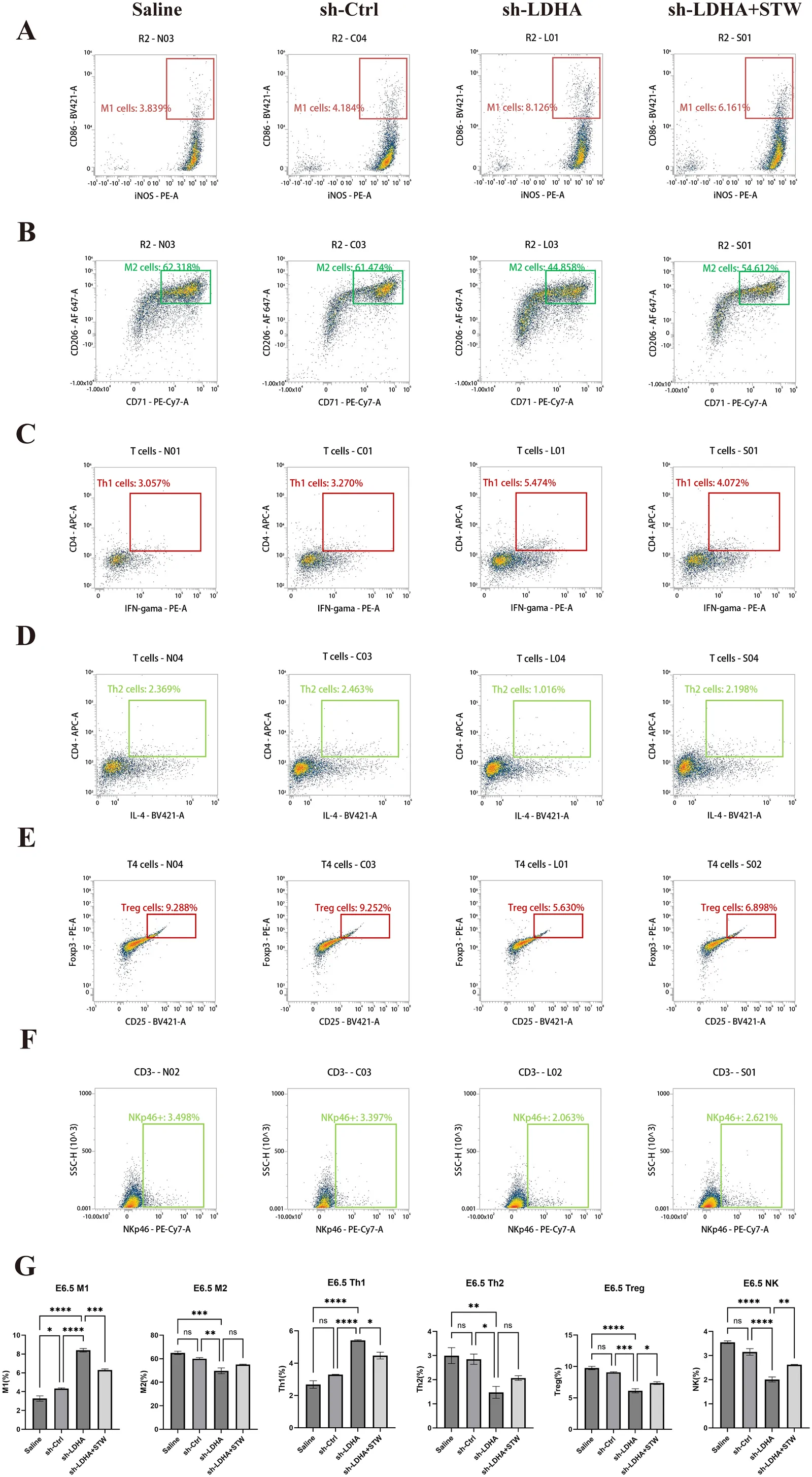
STW modulates the immune cell profile at the maternal-fetal interface on GD 6.5. **(A–F)** Representative flow cytometry plots showing the populations of M1, M2, Th1, Th2, Treg, and NK cells across experimental groups. **(G)** Quantitative analysis of absolute cell numbers per group. Data are expressed as mean ± SEM. n = 3 mice per group for flow cytometry. *P < 0.05, **P < 0.01, ***P < 0.001, ****P < 0.0001. The symbol "ns" denotes not significant.

**Figure 7:**
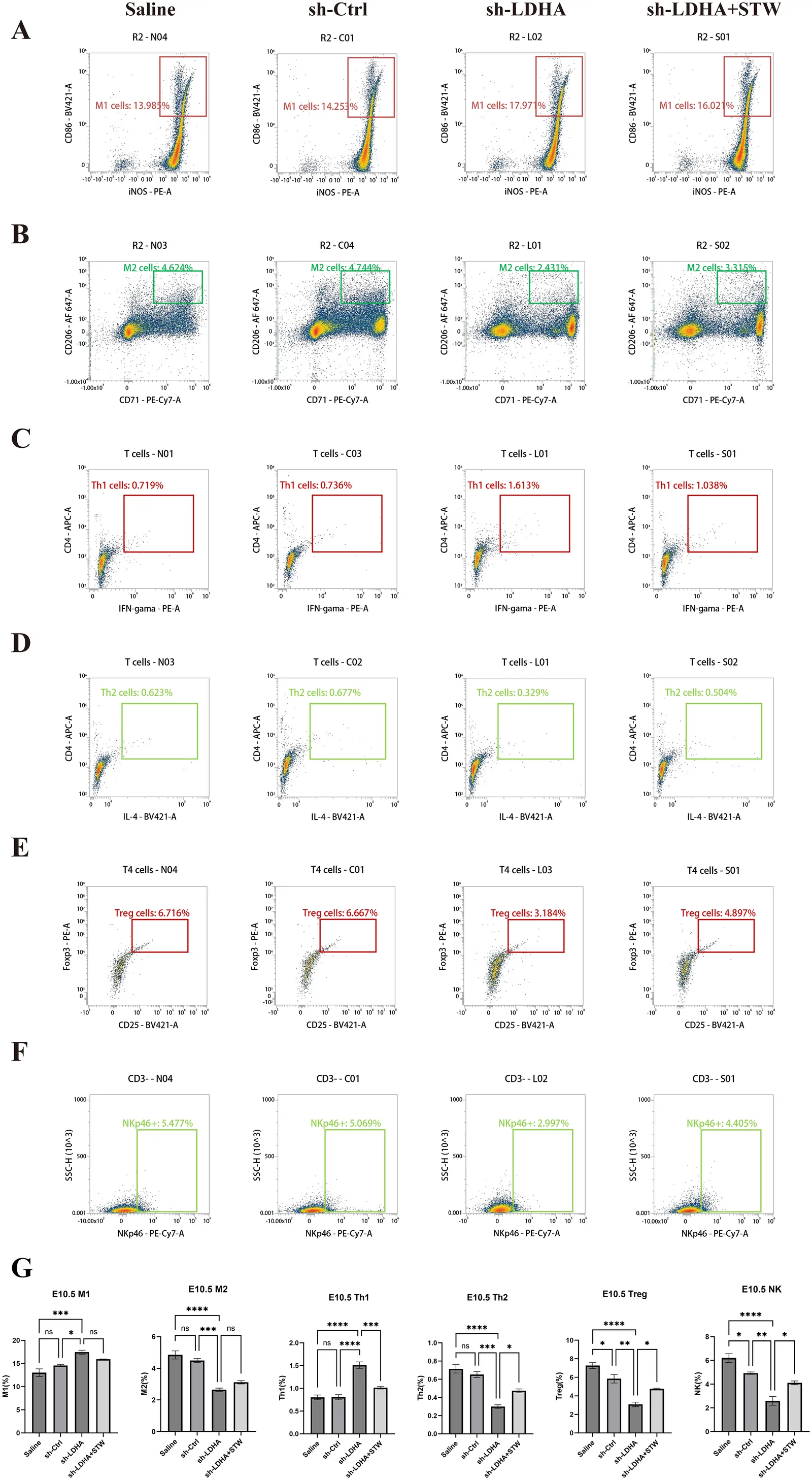
STW modulates the immune cell profile at the maternal-fetal interface on GD 10.5. **(A–F)** Representative flow cytometry plots showing the populations of M1, M2, Th1, Th2, Treg, and NK cells across experimental groups. **(G)** Quantitative analysis of absolute cell numbers per group. Data are expressed as mean ± SEM. n = 3 mice per group for flow cytometry. *P < 0.05, **P < 0.01, ***P < 0.001, ****P < 0.0001. The symbol "ns" denotes not significant.

To further quantify uterine immune cell subsets, flow cytometry was performed across all groups. At GD 6.5 and 10.5, flow cytometry showed a pro-inflammatory shift in *sh-Ldha* mice, characterized by increased M1 and Th1 populations and decreased M2, Th2, Treg, and NK cells compared to controls. STW treatment partially reversed this imbalance. At GD 6.5, STW reduced Th1 and M1 levels and increased Treg and NK counts, while Th2 and M2 levels remained unchanged. By GD 10.5, STW significantly reduced Th1 cells and elevated Th2, Treg, and NK populations, while M1 and M2 counts showed no significant changes (**Figures 6**, **7**).

**Figure 8:**
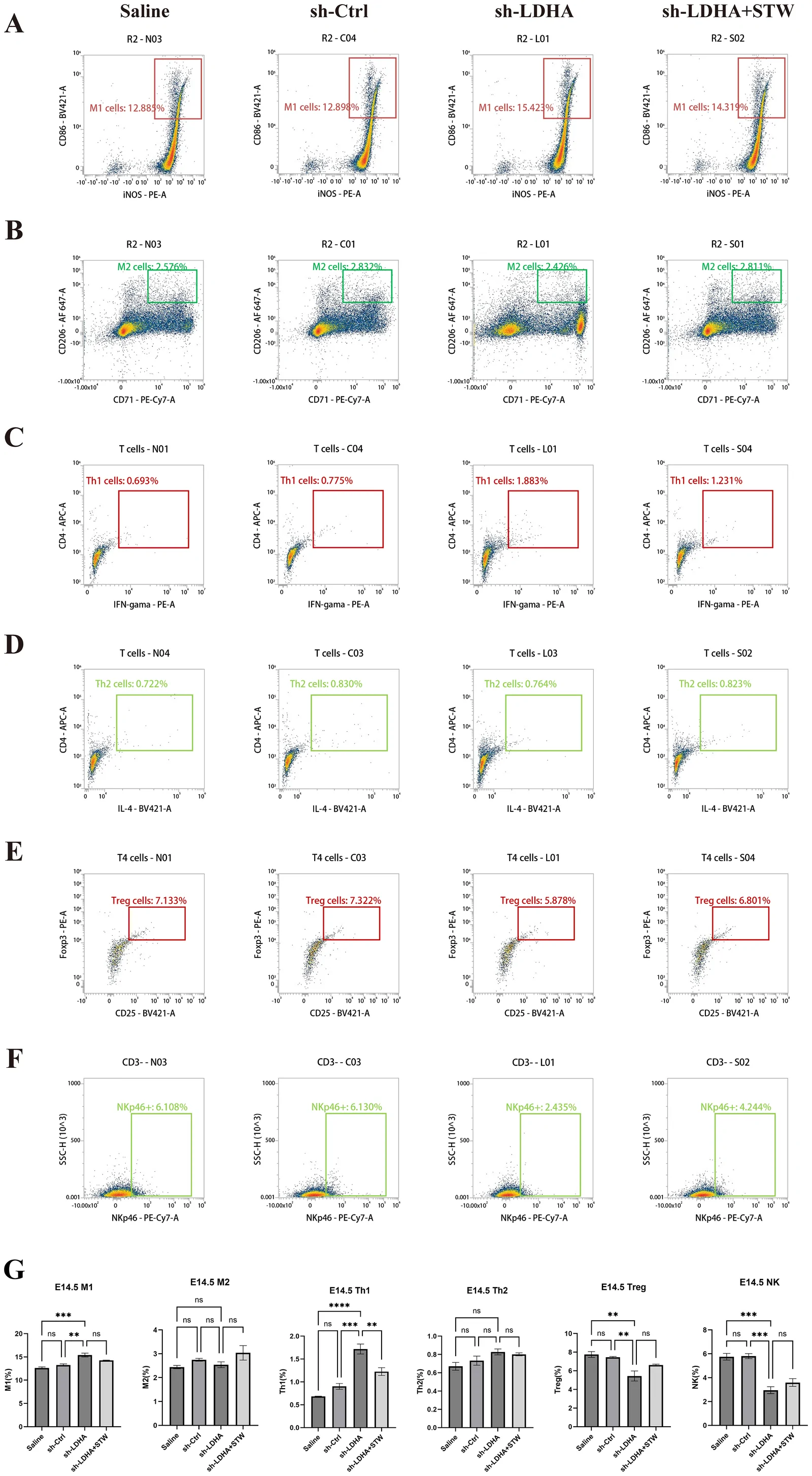
STW modulates the immune cell profile at the maternal-fetal interface on GD 14.5. **(A–F)** Representative flow cytometry plots showing the populations of M1, M2, Th1, Th2, Treg, and NK cells across experimental groups. **(G)** Quantitative analysis of absolute cell numbers per group. Data are expressed as mean ± SEM. n = 3 mice per group for flow cytometry. **P < 0.01, ***P < 0.001, ****P < 0.0001. The symbol "ns" denotes not significant.

At GD 14.5, the *sh-Ldha* group continued to show elevated Th1/M1 and reduced Treg/NK levels. At this stage, STW primarily modulated the T-cell profile, significantly decreasing Th1 infiltration without markedly altering Th2, Treg, NK, or macrophage (M1/M2) subsets relative to the *sh-Ldha* group (**Figure 8**).

### 3.6 STW modulates cytokines expression at the maternal-fetal interface in sh-*Ldha* mice

Next, we evaluated the expression of key immune factors following uterine LDHA inhibition. At GD 6.5 and 10.5, IL-4 and Foxp3 expression (mRNA and protein) declined significantly in the *sh-Ldha* group compared to Saline and sh-Ctrl groups, whereas IFN-γ levels were markedly elevated. Compared to the *sh-Ldha* group, STW up-regulated IL-4 and TGF-β1 at GD 6.5 and maintained higher TGF-β1 levels at GD 10.5. By GD 14.5, *sh-Ldha* mice showed persistent deficits in IL-4, Foxp3, and TGF-β1, while STW significantly suppressed IFN-γ expression. No notable differences were found between the Saline and sh-Ctrl groups throughout the three gestational stages (**Figures 1F–N**).

## 4 Discussion

Successful pregnancy depends on a precise immune-tolerant microenvironment established by uterine immune cells to protect the developing fetus. In mice, decidualization begins as the trophectoderm adheres to the uterine epithelium around GD 4.5–5.5 (44). By GD 6.5, the immune cell network at the maternal-fetal interface matures alongside decidual cells, when the uterine environment transitions from a pro-inflammatory to an immune-tolerant state (45). GD 10.5 marks the start of mid-pregnancy, characterized by complete decidualization, placental formation, and stable immune tolerance (46). By GD 14.5, the functional placenta and expanded maternal blood sinuses support rapid fetal growth (47). Therefore, we selected these three time points to evaluate the impact of uterine LDHA on immune regulation at the maternal-fetal interface.

Intrauterine injection of AAV9-*Ldha*-RNAi significantly downregulated uterine LDHA expression and reduced embryo numbers, consistent with findings in RSA mouse models (27, 48, 49). Morphological analysis confirmed that LDHA deficiency disrupted late-stage placental labyrinth structures and blood supply, both of which were normalized by STW treatment. These findings indicate that inhibiting this key aerobic glycolytic enzyme impairs pregnancy outcomes, while STW restores LDHA expression and improves these results. Interestingly, the lack of obvious embryo resorption suggests that uterine LDHA deficiency may impair early development. Given that inhibiting embryonic LDHA reduces lactate and histone lactylation to slow pre-implantation growth (50), we speculate that uterine LDHA suppression adversely affects embryos before implantation, leading to failure.

Flow cytometry showed M2 predominance at GD 6.5, reflecting an anti-inflammatory uterine state. However, contrary to expectations, M1 macrophages outnumbered M2 cells at GD 10.5 and GD 14.5. While M2/Th2-mediated immune tolerance is vital for pregnancy, M1 macrophages at the maternal-fetal interface are non-redundant. Mid-pregnancy in mice is critical for placental function and uterine spiral artery remodeling. A mild inflammatory environment, driven by M1 macrophage-derived pro-inflammatory cytokines, is essential for clearing apoptotic vascular smooth muscle cells and promoting trophoblast invasion. Supporting this, clinical uterine artery Doppler studies revealed that decidual macrophages from women at high risk for preeclampsia secreted lower IL-1β and TNF-α levels, with culture supernatants failing to promote SGH-PL4 invasion (51). By GD 14.5, when placental blood flow peaks, a higher proportion of M1 macrophages may prevent maternal pathogens from crossing the placental barrier (52). It should also be considered whether the surgical procedure itself—specifically, the intrauterine injection and dorsal incision—may have influenced the uterine immune microenvironment. Surgical trauma can induce localized inflammatory responses that potentially affect decidual immune cell composition and function. However, several observations argue against a major confounding effect of surgery in this study. First, the sh-Ctrl group, which underwent the same surgical procedure and received control AAV9 virus, showed no significant differences from the Saline group in embryo numbers, placental morphology, or immune cell populations at any gestational time point. Second, the immune alterations observed in sh-Ldha mice were specific to LDHA knockdown and were reversed by STW treatment, rather than reflecting a persistent surgical effect. Nevertheless, we cannot completely exclude the possibility that surgery-induced inflammation may have subtly modified the uterine microenvironment, and future studies using non-surgical gene delivery methods or refined minimally invasive techniques would help clarify this issue.

During pregnancy, dNK cells maintain immune tolerance and promote uterine remodeling (7, 53) by secreting pleiotrophin, osteoglycin and other growth factors (8, 9). Post-implantation, dMφs polarize to an M2 phenotype, secreting TGF-β and IL-10 to maintain immune tolerance, clear apoptotic cells, and support spiral artery remodeling (54). While not dominant cell population, decidual T lymphocytes are critical for early maternal-fetal tolerance and are linked to RSA (55). Naïve CD4^+^ T cells differentiate into Th and Treg subsets upon stimulation, an increased Th1/Th2 or Treg/Th17 ratios can disrupt uterine immune balance (56, 57). During implantation, Th1 cells secrete IFN-γ and TNF-α to facilitate moderate trophoblast invasion (58). After implantation, the immune environment shifts toward a Th2 bias, in which TGF-β and IL-4 suppress inflammation and promote fetal growth. In RSA patients, elevated cytotoxic CD8^+^ T cells secrete perforin and granzymes, potentially causing early miscarriage (59). Likewise, activated CD11c^+^CD8^+^ T cells in control mice reduce offspring survival and trophoblast invasion to RSA model levels (60).

We performed IHC staining to identify CD4^+^ T, CD8^+^ T, Mφ, and NK cells at the maternal-fetal interface. At GD 6.5, STW treatment upregulated CD4^+^ T cells and inhibited CD8^+^ T cells in *sh-Ldha* mice. However, at GD 10.5 and 14.5, immune cell distribution was sparser than at GD 6.5, with no significant differences between groups. Previous studies suggests that macrophages at the maternal-fetal interface are gradually replaced by neutrophils as gestation proceeds (61). We thus speculate that the expansion of placental and interstitial cells dilutes immune cell density per unit area. Moreover, after GD 10.5, rapid embryonic growth and placental maturation thin the highly active decidual tissue observed at GD 6.5. Flow cytometry quantified immune cell subsets. In *sh-Ldha* mice, M1 and Th1 cells remained elevated throughout pregnancy, while M2, Th2, Treg, and NK cells were reduced, indicating a disrupted immune-tolerant microenvironment. STW exhibited varying degrees of regulatory effects on uterine immune cell populations, contributing to restoration of immune tolerance.

We also examined the impact of STW on key immune factors at the maternal-fetal interface in *sh-Ldha* mice. Interleukin-4 (IL-4) and interferon-γ (IFN-γ) are the signature cytokines for Th2 and Th1 cells, respectively. Although moderate IFN-γ facilitates trophoblast apoptosis to regulate invasion, its overexpression drives potent pro-inflammatory effects associated with adverse pregnancy outcomes (62). IL-4 regulates the Th1/Th2 balance and supports Treg function to suppress uterine inflammation (63). Consistent with findings that RSA models exhibit elevated Th1 and reduced Th2 cytokines, an increased IFN-γ/IL-4 ratio serves as a sensitive biomarker for diagnosing URPL and predicting pregnancy success (19, 64, 65). Forkhead Box P3 (Foxp3), a well-recognized master regulator of Treg development and function, is stimulated by progesterone, which promotes Foxp3^+^ Treg proliferation and TGF-β1 expression. CD4^+^CD25^+^Foxp3^+^ Tregs are highly specific markers for predicting miscarriage risk (66, 67). In URPL patients, reduced Treg numbers and disordered Foxp3 promoter methylation lead to impaired Foxp3 synthesis and loss of immune suppression (68, 69). Furthermore, TGF-β1, the primary inhibitory cytokine secreted by Treg cells, is significantly lower in RSA patients than in healthy pregnant women, making it a valuable prognostic indicator (70, 71). Our results demonstrate that throughout pregnancy, *sh-Ldha* mice exhibited lower anti-inflammatory and higher pro-inflammatory cytokine levels compared to controls. STW treatment upregulated anti-inflammatory factors and inhibited IFN-γ expression in *sh-Ldha* mice, thereby mitigating the excessive pro-inflammatory response at the maternal-fetal interface.

Our previous studies demonstrated that insufficient aerobic glycolysis in the decidua and trophoblasts of RSA mice reduces lactate production, disrupting the acidic environment of the maternal-fetal interface and impairing embryonic development (27, 48, 49), including the breakdown of immune tolerance (34). This study links STW-mediated aerobic glycolysis to immune balance in RSA mice. *Sh-Ldha* mice mirrored the RSA pro-inflammatory state, showing abnormal immune factors and cell counts. By upregulating LDHA, STW restored these parameters to an anti-inflammatory state. These findings emphasized the necessity of uterine LDHA for pregnancy and clarify how STW promotes immune tolerance by glycolytic metabolism. The dosage of STW used in this study (0.15 g/10 g/day, equivalent to twice the human clinical dose) was selected based on our previous dose-response experiments in RSA mice, in which this dosage exhibited the most consistent effect on restoring uterine glycolytic activity and improving embryo survival (27). While a comprehensive dose-response curve was not repeated in the present uterine-specific knockdown model, the reproducibility of STW’s effects across multiple independent cohorts—including our previous studies using systemic RSA models (27, 31, 48, 49)—supports the appropriateness of this dosage. Nevertheless, we acknowledge that the optimal STW dosage may vary depending on the etiology and severity of pregnancy loss, and future studies are warranted to explore dose-response relationships in different RSA models.

Beyond LDHA-mediated glycolysis, multiple factors have been shown to disturb the decidual immune microenvironment and contribute to adverse pregnancy outcomes. Accumulating evidence indicates that dysregulated placental function, aberrant inflammatory signaling, and altered immune cell trafficking are interconnected processes that collectively determine pregnancy maintenance (72, 73). For instance, an imbalanced sFlt-1/PlGF ratio—a hallmark of placental vascular dysfunction—has been linked to the pathogenesis of preeclampsia (72), while impaired trophoblast survival and function have been associated with adverse pregnancy outcomes (73). These observations suggest that the immune-metabolic crosstalk at the maternal-fetal interface is complex and likely involves intersecting pathways beyond LDHA alone. Moreover, the clinical association between placental structural integrity and pregnancy outcomes underscores the importance of maintaining both metabolic sufficiency and immune homeostasis for successful gestation. Our findings that LDHA knockdown disrupts placental labyrinth architecture and immune cell balance, and that STW restores both, add to this growing body of evidence by highlighting the central role of glycolytic metabolism in integrating placental function with immune regulation. Future studies integrating multi-omics approaches—including metabolomics, transcriptomics, and immune profiling—will be essential to delineate the full network of interactions between metabolism and immunity at the maternal-fetal interface.

From a clinical perspective, our findings have translational implications for the management of recurrent spontaneous abortion. The observation that uterine LDHA knockdown recapitulates key immunological features of RSA—namely, elevated Th1/M1 responses and reduced Treg/NK populations—suggests that metabolic dysfunction at the maternal-fetal interface may be a contributing factor in at least a subset of URPL patients. While direct measurement of LDHA activity in human decidual tissue is challenging, the development of non-invasive or minimally invasive biomarkers reflecting glycolytic activity (e.g., lactate levels in uterine fluid or circulating metabolic intermediates) could potentially aid in risk stratification and guide therapeutic decisions. Furthermore, the efficacy of STW in restoring immune balance in the LDHA-deficient model supports its continued use as an adjunctive therapy in RSA, particularly in patients with evidence of metabolic or immune dysregulation. However, it is important to note that the translation of these findings to human pregnancy requires caution, as the timing and regulation of decidualization, placentation, and immune cell trafficking differ between mice and humans. Future clinical studies correlating decidual LDHA expression, lactate levels, and immune cell profiles in RSA patients will be essential to validate the clinical relevance of the LDHA-lactate-immune axis identified in this study.

Several limitations of this study should be acknowledged. First, although our previous studies have demonstrated that STW restores lactate levels in RSA mouse models (27, 31, 48, 49), the present study did not directly measure lactate concentrations or local pH at the maternal-fetal interface in the uterine-specific LDHA knockdown model. Given that the patterns of metabolic alteration in this model may differ from those in systemic RSA models, direct measurement of lactate and pH would provide more definitive evidence linking uterine LDHA knockdown to disruption of the acidic microenvironment. Nevertheless, the consistent reduction in LDHA expression (both mRNA and protein) across all three gestational time points, together with the parallel changes in immune cell populations and cytokine profiles that align with the expected consequences of lactate deficiency, supports the mechanistic interpretation that LDHA knockdown impairs immune tolerance through reduced glycolytic activity. Future studies incorporating *in situ* lactate detection and pH measurements are warranted to directly validate this pathway. Second, in the flow cytometric analysis of macrophage subsets, we identified M1- and M2-like cells based solely on CD86/iNOS and CD206/CD71 expression without prior lineage gating for F4/80^+^CD11b^+^ macrophages. While the markers used are well-established for macrophage polarization states, the absence of a macrophage-lineage gate means that the populations we quantified are best interpreted as “M1-like” and “M2-like” cells rather than definitive M1/M2 macrophages. This distinction does not undermine our major conclusions—given the consistent trends across IHC (F4/80^+^ staining), Western blot, and qRT-PCR—but should be considered when comparing our flow cytometric data with studies that employed full-lineage gating. Future studies will incorporate F4/80/CD11b co-staining to confirm these findings. Third, although viral transduction efficiency was not directly visualized by immunofluorescence or EGFP fluorescence microscopy, the significant and sustained suppression of LDHA protein and mRNA across three gestational time points confirms the reliability of our uterine-specific intervention. The AAV9 vector used in this study carries an EGFP reporter gene (U6-MCS-CAG-EGFP), and the consistent knockdown effect observed at all time points indirectly supports adequate transduction of uterine tissues. Moreover, the absence of systemic toxicity or overt off-target effects, as reflected by normal maternal behavior and weight gain in the sh-Ldha group, suggests that viral leakage to other organs, if any, was minimal. Nonetheless, we acknowledge that direct visualization of EGFP expression or detection of LDHA levels in non-uterine tissues (e.g., liver, kidney, and ovary) would provide more definitive evidence of uterine specificity, and this should be addressed in future studies.

While STW broadly promotes aerobic glycolysis at the maternal-fetal interface (27, 29), this study focused exclusively on LDHA. Future research should investigate other key glycolytic enzymes, such as PKM2 and HK2, to provide a more comprehensive mechanistic landscape of how STW regulates maternal-fetal metabolism.

## Data Availability

The original contributions presented in the study are included in the article/**Supplementary Material**. Further inquiries can be directed to the corresponding author.

## Supplementary material

Supplementary Table 6 Flow cytometry gating strategies for immune cell subsets.
