# Supplementary material for: Shoutai Wan regulates maternal-fetal immune tolerance in lactate dehydrogenase A knockdown model mice to prevent miscarriage
Source: Front Immunol. 2026 Sep 9;17:1885151. doi: 10.3389/fimmu.2026.1885151 (PMC13597236; doi:10.3389/fimmu.2026.1885151)
Supplement: Supplementary file 1 [file SupplementaryFile1.docx]

**Supporting Information**

**Title:** Shoutai Wan regulates maternal-fetal immune tolerance in lactate dehydrogenase A knockdown model mice to prevent miscarriage

**Authors:** Ziwei Zhou ^#^, Yuhan Du ^#^, Ruigan Shi, Siling Tang, Mengmeng Zhou, Huilan Du *

* Corresponding author. ^#^ Contributed equally.

**This file includes:**

Supplementary Table S1: List of Abbreviations

Supplementary Table S2: The composition of Shoutai Wan

Supplementary Table S3: Antibodies information

Supplementary Table S4: Reagents information

Supplementary Table S5: Primers sequence

**Table S1. List of Abbreviations**

| **Abbreviation** | **Full name** |
| --- | --- |
| STW | Shoutai Wan |
| GD | gestational day |
| IHC | Immunohistochemical |
| qRT-PCR | Quantitative Reverse Transcription Polymerase Chain |
| WB | Western blot |
| FACS | Fluorescence-activated cell sortin |
| NK | Natural killer cell |
| Mφ | Macrophages |
| Treg | Regulatory T cells |
| Th | Helper T cells |
| LDHA | Lactate Dehydrogenase A |
| IL-4 | Interleukin-4 |
| TGF-β1 | Transforming growth factor beta 1 |
| Foxp3 | Forkhead box protein 3 |
| IFN-γ | Interferon gamma |

**Table S2. The composition of Shoutai Wan**

| **Chinese herbs** | **Latin name** | ****Scientific Name (Latin Binomial)**** | ****Family**** | ****Part Used**** | ****Proportion (g)**** |
| --- | --- | --- | --- | --- | --- |
| Tu Si Zi | *Cuscutae Semen* | *Cuscuta chinensis Lam.* | Convolvulaceae | Seed | 20 |
| Chuan Xu Duan | *Dipsaci Radix* | *Dipsacus asper Wall. ex DC.* | Caprifoliaceae | Root | 10 |
| Sang Ji Sheng | *Taxilli Herba* | *Taxillus chinensis (DC.) Danser* | Loranthaceae | Herb (stem and leaf) | 10 |
| E Jiao | *Colla Corii Asini* | *Colla Corii Asini* | Equidae | Donkey-hide glue | 10 |

**Table S3. Antibodies information**

| **Target** | **Clone** | **Host/Isotype** | **Application** | **Dilution** | **Vendor** | **Catalog No.** |
| --- | --- | --- | --- | --- | --- | --- |
| CD4 | D7D2Z | Rabbit IgG | IHC | 1:100 | Cell Signaling Technology | #25229 |
| CD8α | D4W2Z | Rabbit IgG | IHC | 1:100 | Cell Signaling Technology | #98941 |
| F4/80 | 81668-1-RR | Rabbit IgG | IHC | 1:8000 | Proteintech | #81668-1-RR |
| CD49a | PSH14-05 | Rabbit IgG | IHC | 1:200 | HUABIO | #HA723595 |
| CD49b | R09-7C3 | Rabbit IgG | IHC | 1:100 | Zenbio | #R381338 |
| CD86 | GL1 | Rat (LOU) IgG2a, κ | FACS | 5 μL/test | BD Biosciences | #564198 |
| CD206 | MR5D3 | Rat (F344) IgG2a | FACS | 5 μL/test | BD Biosciences | #565250 |
| iNOS | W16030C | Rat IgG2b, κ | FACS | 2 μL/test | Biolegend | #696806 |
| CD71 | RI7217 | Rat IgG2a, κ | FACS | 5 μL/test | Biolegend | #113812 |
| CD3-FITC | 145-2C11 | Armenian Hamster IgG1, κ | FACS | 5 μL/test | BD Biosciences | #561827 |
| CD4-APC | RM4-5 | Rat (DA) IgG2a, κ | FACS | 5 μL/test | BD Biosciences | #553051 |
| IFN-γ-PE | XMG1.2 | Rat IgG1, κ | FACS | 5 μL/test | BD Biosciences | #554412 |
| IL-4-BV421 | 11B11 | Rat IgG1, κ | FACS | 5 μL/test | BD Biosciences | #562915 |
| CD25-BV421 | 3C7 | Rat (LEW) IgG2b, κ | FACS | 5 μL/test | BD Biosciences | #564370 |
| Foxp3-PE | R16-715 | Rat IgG2a, κ | FACS | 5 μL/test | BD Biosciences | #563101 |
| CD335-PE Cy7 | 29A1.4 | Rat IgG2a, κ | FACS | 5 μL/test | eBioscience | #25-3351-82 |
| LDHA | EP1566Y | Rabbit IgG | WB | 1:1000 | abcam | #ab52488 |
| TGF-β1 | P01137 | Rabbit IgG | WB | 1:1000 | Proteintech | # 21898-1-AP |
| Foxp3 | [Q9BZS1](https://www.uniprot.org/uniprot/Q9BZS1) | Rabbit IgG | WB | 1:500 | Proteintech | #22228-1-AP |
| IL-4 | P05112 | Mouse IgG2a | WB | 1:500 | Proteintech | #366142-1-Ig |
| IFN-γ | P01580 | Rabbit IgG | WB | 1:500 | Immunoway | #YT2279 |
| β-actin | [P60709](https://www.uniprot.org/uniprot/P60709) | Rabbit IgG | WB | 1:6000 | Proteintech | #20536-1-AP |
| Beta tubulin | Q13509 | Rabbit IgG | WB | 1:5000 | Proteintech | #10094-1-AP |
| Goat anti-rabbit IgG | - | Goat | WB | 1:5000 | Zenbio | #511203 |
| Goat anti-mouse IgG | - | Goat | WB | 1:5000 | Zenbio | #511103 |

**Table S4. Reagents information**

| **Reagent** | **Application** | **Dilution** | **Vendor** | **Catalog No.** |
| --- | --- | --- | --- | --- |
| EDTA buffer | IHC | 1:20 | Servicebio | # G1203-250ML |
| Endogenous peroxidase blocker | IHC | - | ZSGB-BIO | # PV-9001 |
| reaction enhancement solution | IHC | - | ZSGB-BIO | # PV-9001 |
| enhanced enzyme-labeled goat anti-rabbit IgG polymer | IHC | - | ZSGB-BIO | # PV-9001 |
| DAB chromogen | IHC | 1:20 | ZSGB-BIO | # ZLI-9018 |
| RPMI 1640 medium | FACS | - | Hyclone | # SH30027.02 |
| fixation/permeabilization reagent | FACS | - | BD | # 554714 |
| cell stimulation cocktail (containing protein transport inhibitors) | FACS | 1:500 | eBioscience | # 00-4975-93 |
| fixation/permeabilization buffer | FACS | 1:3 | eBioscience | # A530170 |
| protein lysis buffer | WB | - | Bestbio | # BB-3201 |
| BCA kit | WB | - | Seven biotech | # SW101-02 |
| 5 × Loading Buffer | WB | - | Applygen | # B1012 |
| PVDF membranes | WB | - | Millipore | # ISEQ00010 |
| chemiluminescent substrate | WB | - | Abbkine | # BMU102 |
| Total RNA Kit II | qRT-PCR | - | Omega | # R6934-01 |
| cDNA Synthesis SuperMix | qRT-PCR | - | novoprotein | # E047 |
| 2 × SYBR Green qPCR Master Mix | qRT-PCR | - | Servicebio | # G3321 |

**Table S5. Primers sequence**

| **Target Gene** | **Forward Primer (5’-3’)** | **Reverse Primer (5’-3’)** | **Product Size (bp)** |
| --- | --- | --- | --- |
| *Ldha* | AAGTTTACACGAAGGTCGACAT | ACTTGAAGATGTTCACGTTTCG | 157 |
| *Tgf-β1* | TCAGACATTCGGGAAGCAGT | ACGCCAGGAATTGTTGCTAT | 47 |
| *Il-4* | CAGCTAGTTGTCATCCTGCTCTTC | TCCCTTCTCCTGTGACCTCGTT | 118 |
| *Foxp3* | ATGAGAAAGGCAAGGCCCAGT | GTGGCTACGATGCAGCAAGA | 181 |
| *Ifn-γ* | TGAACGCTACACACTGCATCTTG | TCCACATCTATGCCACTTGAGTT | 133 |
| *β-actin* | GTGACGTTGACATCCGTAAAGA | GTAACAGTCCGCCTAGAAGCAC | 287 |
