# Supplementary material for: Shoutai Wan regulates maternal-fetal immune tolerance in lactate dehydrogenase A knockdown model mice to prevent miscarriage
Source: Front Immunol. 2026 Sep 9;17:1885151. doi: 10.3389/fimmu.2026.1885151 (PMC13597236; doi:10.3389/fimmu.2026.1885151)
Supplement: Supplementary file 2 [file Table6.docx]

**Supporting Materials**

**Title: Shoutai Wan regulates maternal-fetal immune tolerance in lactate dehydrogenase A knockdown model mice to prevent miscarriage**

**Authors:** Ziwei Zhou ^#^, Yuhan Du ^#^, Ruigan Shi, Siling Tang, Mengmeng Zhou, Huilan Du *

* Corresponding author. ^#^ Contributed equally.

**This file includes:**

Supplementary Table 6-1: Gating strategy for labeling M1-like and M2-like cells by flow cytometry

Supplementary Table 6-2: Gating strategy for labeling Th1 and Th2 cells by flow cytometry

Supplementary Table 6-3: Gating strategy for labeling Treg and NK cells by flow cytometry

**Gating strategy for flow cytometry**

1. Gating strategy for labeling M1‑like and M2‑like cells in mouse uterus with conceptus tissues

a) Intact main cell populations with normal morphology were initially gated using SSC‑H (10³) versus FSC‑H (10³).

b) Doublets within the target cell population were excluded using FSC‑H (10³) versus FSC‑A (10³).

c) M1‑like cells in the target population were labeled with CD86‑BV421‑A and iNOS‑PE‑A; M2‑like cells in the target population were labeled with CD206‑AF647‑A and CD71‑PE‑Cy7‑A.

Note: No commercial viability dyes were used in this protocol to label live or dead cells. Instead, live and dead cells were discriminated based on their intrinsic differences in forward scatter (FSC) and side scatter (SSC) signals. Throughout the experiment, intact main cell populations with normal morphology were gated on FSC‑SSC dot plots, and fragmented or dead cells with low SSC and low FSC signals were removed to achieve dead‑cell exclusion.

The complete gating strategy is summarized below:

**Table S6-1. Gating strategy for labeling M1-like and M2-like cells by flow cytometry**

| **Gating Step** | **Population** | **Rationale** |
| --- | --- | --- |
| SSC-H/FSC-H | Main cell population | Morphological selection |
| FSC-H/FSC-A | Single cell | Doublet exclusion |
| CD86/iNOS | M1-like cells | Subpopulation |
| CD206/CD71 | M2-like cells | Subpopulation |

2. Gating strategy for labeling Th1 and Th2 cells in mouse uterus with conceptus tissues

a) Intact main cell populations with normal morphology were initially gated using SSC‑H (10³) versus FSC‑H (10³).

b) Doublets within the target cell population were excluded using FSC‑H (10³) versus FSC‑A (10³).

c) T lymphocytes within the target cell population were labeled using SSC‑H (10³) versus CD3‑FITC‑A.

d) Th1 cells were labeled with CD4‑APC‑A and IFN‑gamma‑PE‑A; Th2 cells were labeled with CD4‑APC‑A and IL‑4‑BV421‑A.

The complete gating strategy is summarized below:

**Table S6-2. Gating strategy for labeling Th1 and Th2 cells by flow cytometry**

| **Gating Step** | **Population** | **Rationale** |
| --- | --- | --- |
| SSC-H/FSC-H | Main cell population | Morphological selection |
| FSC-H/FSC-A | Single cell | Doublet exclusion |
| SSC-H/CD3 | T lymphocytes | T-cell lineage confirmation |
| CD4/IFN-gamma | Th1 cells | T-cell subpopulations |
| CD4/IL-4 | Th2 cells | T-cell subpopulations |

3. Gating strategy for labeling Treg and NK cells in mouse uterus with conceptus tissues

a) Intact main cell populations with normal morphology were initially gated using SSC‑H (10³) versus FSC‑H (10³).

b) Doublets within the target cell population were excluded using FSC‑H (10³) versus FSC‑A (10³).

c) T lymphocytes within the target cell population were labeled using SSC‑H (10³) versus CD3‑FITC‑A.

d) Within the CD3⁺ cell population, helper T‑cell subpopulations were labeled using SSC‑H (10³) versus CD4‑APC‑A.

e) Treg cells were labeled with Foxp3‑PE‑A and CD25‑BV421‑A.

f) Within the CD3⁻ cell population, NK cells were labeled using SSC‑H (10³) versus NKp46‑PE‑Cy7‑A.

The complete gating strategy is summarized below:

**Table S6-3. Gating strategy for labeling Treg and NK cells by flow cytometry**

| **Gating Step** | **Population** | **Rationale** |
| --- | --- | --- |
| SSC-H/FSC-H | Main cell population | Morphological selection |
| FSC-H/FSC-A | Single cell | Doublet exclusion |
| SSC-H/CD3 | CD3⁺ and CD3⁻ cell populations | T‑cell lineage confirmation |
| (CD3^+^) SSC-H/CD4 | Th cells | T‑cell subpopulations |
| Foxp3/CD25 | Treg cells | T‑cell subpopulations |
| (CD3^-^) SSC-H/NKp46 | NK cells | NK cell markers |
